# The i-Motif as a Molecular Target: More Than a Complementary DNA Secondary Structure

**DOI:** 10.3390/ph14020096

**Published:** 2021-01-27

**Authors:** Susie L. Brown, Samantha Kendrick

**Affiliations:** Biochemistry and Molecular Biology Department, University of Arkansas for Medical Sciences, Little Rock, AR 72205, USA; SBrown6@uams.edu

**Keywords:** i-motif, DNA quadruplex structures, transcription regulation, cancer therapeutics

## Abstract

Stretches of cytosine-rich DNA are capable of adopting a dynamic secondary structure, the i-motif. When within promoter regions, the i-motif has the potential to act as a molecular switch for controlling gene expression. However, i-motif structures in genomic areas of repetitive nucleotide sequences may play a role in facilitating or hindering expansion of these DNA elements. Despite research on the i-motif trailing behind the complementary G-quadruplex structure, recent discoveries including the identification of a specific i-motif antibody are pushing this field forward. This perspective reviews initial and current work characterizing the i-motif and providing insight into the biological function of this DNA structure, with a focus on how the i-motif can serve as a molecular target for developing new therapeutic approaches to modulate gene expression and extension of repetitive DNA.

## 1. Introduction

In 1953, Watson and Crick first published the structure of the DNA double helix [1]. They described a DNA molecule as a right-handed, twisted coil composed of a purine and pyrimidine inner core held together by hydrogen bonds, with a sugar–phosphate backbone that extended from these paired bases. The x-ray diffraction photograph produced by Rosalind Franklin is one of the most readily identifiable images from scientific literature. Initially, two variations of the double helix were identified, the A- and B-forms [2]. These two conformations have distinct positions of the deoxyribose sugar that result in different spacing between nucleotide bases, with DNA most commonly found in the B-form [3]. In the 1970s, Z-DNA, the left-handed duplex, was observed under certain laboratory conditions [4]. This discovery gradually led to the realization that DNA can adopt different configurations. Since this early work uncovering the varied helices of duplex DNA, continued investigations demonstrated that nucleic acids also form sequence-dependent secondary structures, such as triplex DNA, hairpins, and cruciforms, as well as tetraplex structures: the guanine-quadruplex (G4) and the intercalated-motif (i-motif) (reviewed in [5]).

**Figure 1:**
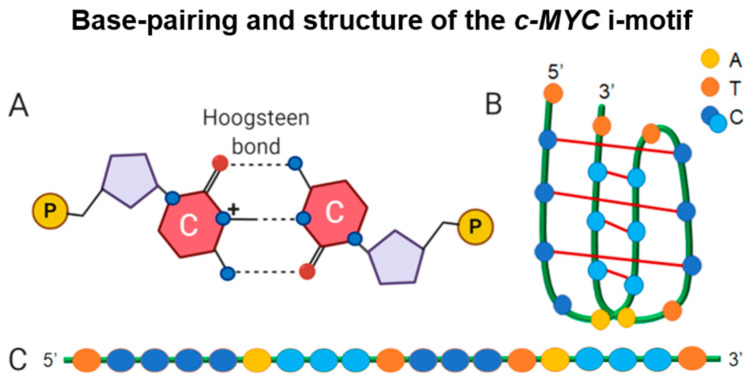
Building blocks of the i-motif structure using *c-MYC* as a model. (**A**) A representation of Hoogsteen base pairing within an i-motif; (**B**) diagram of the *c-MYC* i-motif folded structure; and (**C**) the C-rich sequence that gives rise to an i-motif with the pairing pattern of cytosine tracts illustrated in two different shades of blue [24].

G4 and i-motif structures are formed from stacked tetrads of guanine or cytosine nucleotides, respectively, that can occur in both single strands of DNA and where two or four strands assemble together. G4s were first identified in 1962 and biologically relevant structures in human telomeres were first discovered in 1987 [6,7] to arise from sequences of contiguous guanines stabilized by cations such as K+ [8]. Extensive research has explored the formation of G4 structures, their role in transcriptional regulation and telomere maintenance, and therapeutic potential, particularly in the area of silencing oncogene expression in the treatment of cancer [9,10,11,12,13,14,15]. Nearly 30 years later in 1993, another quadruplex-based DNA structure, the i-motif, was shown in foundational studies using nuclear magnetic resonance (NMR), but unlike the G4, this structure is built from adjacent runs of cytosines [16]. Specifically, the i-motif consists of intercalated hemi-protonated cytosine (C-C+) base pairs with loop regions composed of various bases intervening between the cytosine runs (Figure 1). Similar to the guanines in the G4, base pairing of cytosines occurs via Hoogsteen hydrogen bonds; however, since the formation of i-motif structures also requires the protonation of cytosines, i-motifs form more readily at acidic pH. This pH-dependent formation of i-motifs and the initial lack of evidence that i-motifs could form in solutions at physiological pH led to skepticism within the DNA scientific community as to whether i-motifs existed in vivo. Contrary to the belief that these structures may not form in cellular nuclei, the prevalence of C-rich regions in telomeres and promoter regions suggest that the i-motif serves a biological function [17]. Shortly after the discovery of the i-motif, structures are now recognized to form from C-rich sequences within telomeres as well as those found in the promoters of primarily oncogenes, including *c-MYC* [17]. Beginning in 1997 with i-motif structure formation in the insulin-linked polymorphic region, studies showing that i-motifs have the ability to block DNA replication furthered the idea that i-motifs may regulate nuclear processes [18]. The continuing research pursuits to demonstrate their formation and role in physiological relevant contexts led to several breakthroughs in the i-motif field, particularly in the last few years, and posit these structures as the next likely DNA structure to target for therapeutic development. 

This review focuses on the proposed models for the biological role of i-motifs in the context of select diseases. We discuss how discovery of i-motif ligands serve as both molecular tools to tease out these nuclear functions and potential new therapeutics for drug development. We also highlight the different screening approaches used to identify i-motif interactive agents. For background, we include a brief discussion on the requirements for i-motif formation and the recent detection of these structures in the nuclei of cells, but direct readers to more comprehensive reviews for detailed descriptions of factors affecting i-motif structure and formation [19,20,21,22,23].

## 2. i-Motif Formation and Detection in Living Cells

Even though initial evidence supports the biological relevance of i-motifs, the study of these structures lags behind that of the well-characterized complementary G4 structures. The pH dependency of i-motifs raises the important question of whether these sequences fold into tetraplexes and persist in living cells. Rather than immediate research efforts focused on their biological function, i-motifs received attention as potential molecular switches with applications in nanotechnology, allowing for the development of technologies such as intracellular pH indicators and targeted drug delivery systems [25]. It would take 25 years from the discovery of the i-motif for confirmation of the existence of these structures inside the nuclei of living cells. 

### 2.1. Role of Molecular Crowding, Nucleotide Sequence, and Epigenetic Modification

Demonstration that pH was not the only factor affecting the stability of i-motifs substantially progressed the field and indicated the real possibility these structures may form inside the nucleus. Many factors are now recognized to influence whether a nucleotide sequence is likely to form an i-motif, including the local, molecular environment, loop length and base composition, and epigenetic modification. When molecular crowding agents such as polyethylene glycol (PEG) are included in buffer solutions to mimic the crowded nuclear environment within a cell, the transitional pH for i-motif formation increases to approximately 6.8, substantially closer to physiological levels [26]. It is important to note that the degradation of PEG can lower the pH of a solution, which raises the question as to whether the stabilization effect of PEG is due to this potential change in pH or its molecular crowding effects [27]. Similarly, the torsional strain placed on duplex DNA within the nucleus induces negative superhelicity. When recapitulated in vitro by introducing i-motif sequences into supercoiled plasmids, this negative superhelicity also facilitates the preferential formation of i-motifs in highly C-rich elements over duplex DNA [28]. The specific nucleotide sequence of the i-motif-forming region also impacts the stability of the consequent structure, where, for example, the melting temperature of an i-motif increases with the number of cytosines in sequence [29,30]. Likewise, the length and composition of the loop regions are important and originally, longer lateral loops were thought to permit assembly of more stable structures. However, recent studies show that certain i-motif sequences with shorter loops exhibit increased thermal stability [31]. Longer lateral loops may still contribute to a greater stability due to interactions between specific bases contained within the loop regions. An increased number of cytosines within these regions results in stable structures while a high number of purine bases is associated with decreased stability [32,33]. Evidence also suggests that some longer loop regions form stable hairpins, creating a more stable hybrid i-motif/hairpin structure [34]. Additionally, modifications of the sugar backbone of nucleic acids can alter i-motif stability. Substitution of cytidines with an artificial nucleic acid containing an acyclic scaffold, threoninol, destabilizes i-motifs [35] and 2′-fluoroarabinonucleic acid modifications increase i-motif stability [36]. These modifications provide some control over i-motif stability in laboratory experiments and provide insight into the structure and folding process of i-motifs. Lastly, epigenetic modification of cytosines within i-motif sequences also appears to affect stability. The Waller laboratory examined a range of cytosine modifications and determined that i-motifs stable at physiological pH were more likely to consist of methylated cytosines [37]. The presence of i-motif sequences within CpG islands, which are well-known sites of methylation, supports this finding and indicates that methylation may contribute to i-motif formation within living cells [37,38,39].

### 2.2. Visualization of the i-Motif Using Fluorescent Antibodies and In-Cell NMR

In 2018, Christ et al. identified an antibody fragment, known as iMab, that binds specifically to formed i-motif structures [40]. Their experiments confirmed that the interaction between iMab and i-motifs differentiates between i-motifs and other secondary structures, including G4s. Using three human cancer cell lines, MCF7, U2OS, and HeLa, iMab enables the visualization of formed i-motif structures within telomeres and promoter regions within the nuclei of these living cells. More recently, an antibody against the transcription factor BmILF, which binds with high specificity to the i-motif structure within the promoter region of the gene *BmPOUM2*, further discussed in Section 3.2, detected folded i-motifs in cell nuclei from the testes of the invertebrate, *Bombyx mori* [41]. In support of an active role in gene transcription, the formation of these i-motifs within the testes were cell cycle dependent as indicated by enhanced staining during the G_1_ phase. In addition to the use of antibodies, cutting-edge in-cell NMR also demonstrates that stable, persistent i-motifs form within HeLa cells under physiological conditions [42]. Collectively, these findings confirm what i-motif researchers long suspected, that despite their in vitro dependence on low pH, DNA C-rich sequences can adopt i-motif conformations within the nucleus. 

## 3. i-Motifs Function as Molecular Switches for Gene Regulation

**Figure 2:**
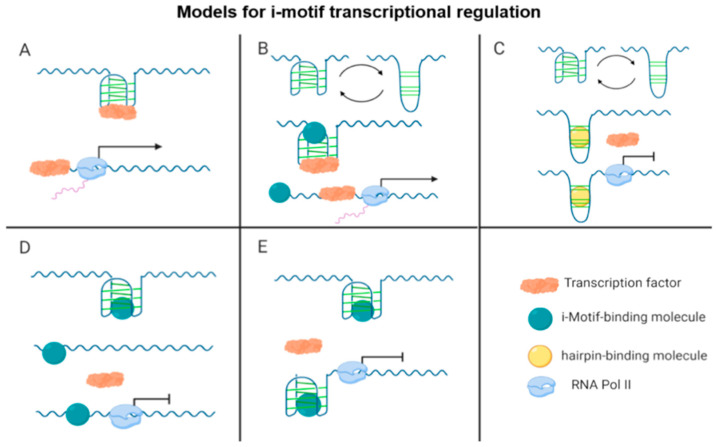
Schematic of the different working models for i-motif function in transcription. (**A**) An i-motif serves as a scaffold for transcription factor binding that leads to i-motif unfolding and transcriptional activation and (**B**–**E**) depict potential effects of i-motif-binding molecules. A small molecule can: (**B**) stabilize the i-motif over a hairpin structure, allowing transcription factor recognition of binding sites on the lateral loops of the i-motif, leading to i-motif unfolding and transcriptional activation; (**C**) bind to a hairpin structure, preventing i-motif formation, resulting in loss of transcription factor binding, and transcriptional repression; (**D**) destabilize the i-motif, leading to i-motif unfolding, loss of transcription factor binding, and transcriptional repression; or (**E**) stabilize the i-motif blocking the transcription factor binding site, leading to transcriptional repression.

Lending further credibility to i-motifs as biologically relevant structures, i-motif-forming sequences are evolutionarily conserved across species, in both prokaryotes and eukaryotes, within telomeres and gene promoter regions [43,44]. Similar to G4s, the close proximity of i-motif elements to transcription start sites infers a role in regulating gene expression. Thus far, data support this cis-regulatory function of the i-motif and indicate the potential to act as an activator and repressor of transcription depending on the specific promoter region, the unique i-motif sequence, and the associated transcription factors (Figure 2). In the human genome, genes encoding oncogenes, bone development proteins, sequence-specific DNA binding domain transcription factors, and molecules related to the positive regulation of transcription from the RNA polymerase II promoter have a high incidence of C-rich sequences capable of forming i-motif structures [45]. Here, we limit our discussion to published work involving three key oncogene i-motif structures as well as an i-motif recently identified from the genome of an insect that offer insight into how i-motifs function as regulators of gene expression. Additional genes are covered in later sections of the review. 

### 3.1. Transcriptional Activator

**Figure 3:**
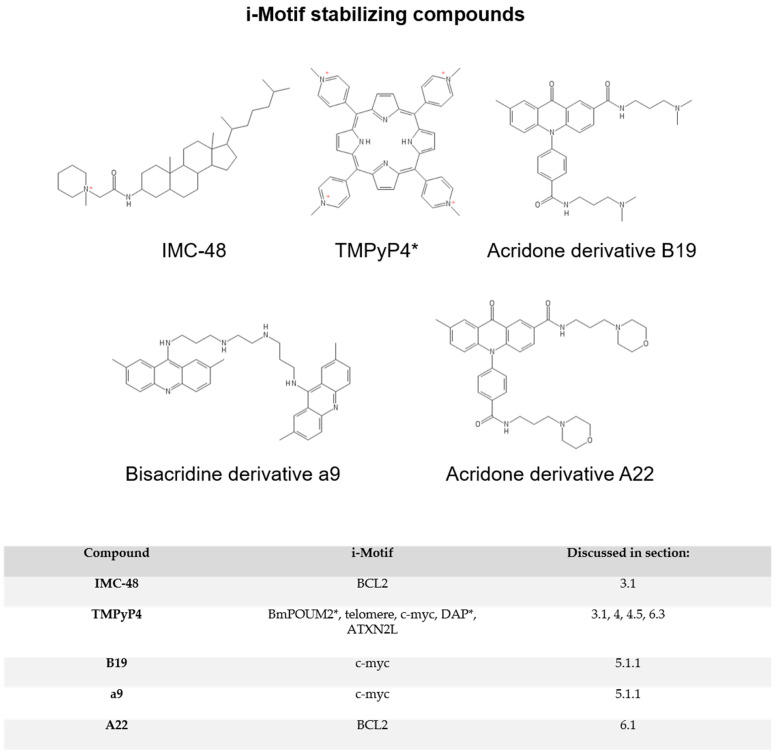
i-Motif stabilizing compounds. * TMPyP4 can also be a destabilizer, depending on the i-motif in question. In the table below, asterisks denote i-motifs destabilized by TMPyP4.

**Figure 4:**
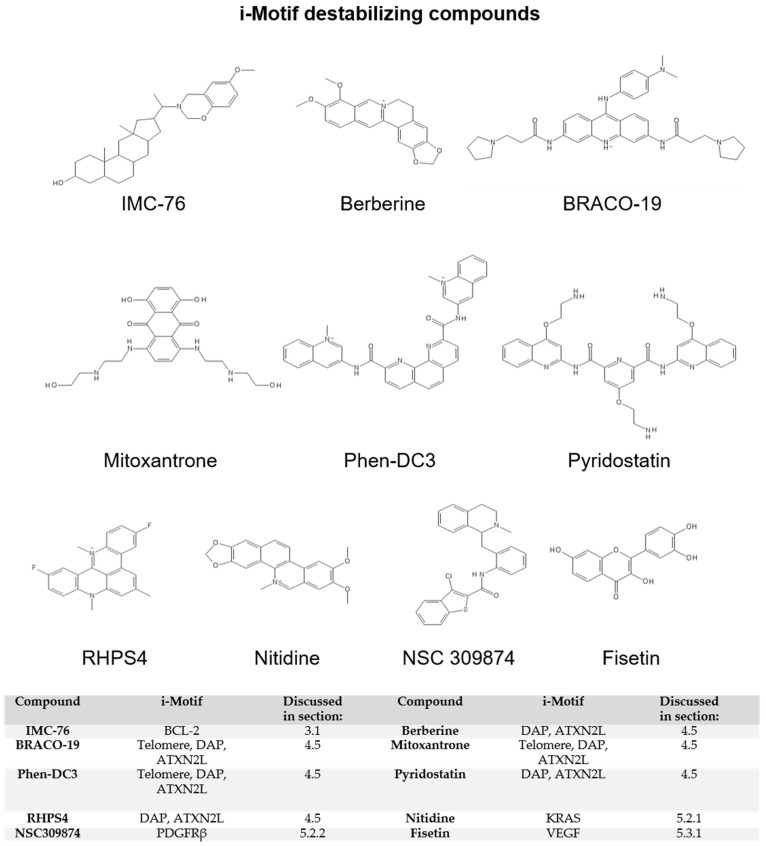
i-Motif destabilizing compounds.

While early research often speculated about the biological relevance of i-motif structures, there was no direct evidence of i-motifs regulating transcription until 2014. Two foundational studies investigating the i-motif-forming sequence within the promoter region of the *BCL2* oncogene demonstrate i-motif elements exhibit a dynamic equilibrium of structures recognized by small molecules analogous to RNA riboswitches [46,47]. *BCL2* encodes an anti-apoptotic protein important in maintaining cell survival and its expression is dysregulated in many cancers, allowing cells to bypass cell cycle checkpoints and contribute to cancer initiation and progression [48]. By screening the NCI Diversity Set library of small molecules, two compounds were identified to interact with the *BCL2* i-motif that either stabilized, IMC-48 (Figure 3), or destabilized the structure, IMC-76 (Figure 4) [46]. The observed destabilization was not as much as an unfolding of the i-motif, but rather a stabilization and trapping out of the hairpin species, thereby preventing i-motif assembly. In contrast, IMC-48 bound to the folded i-motif, presumably at the central loop, and increased stability of the structure. Utilizing these compounds as molecular tools to modulate *BCL2* i-motif formation within B-cell lymphoma and breast cancer cell lines, *BCL2* mRNA expression was substantially reduced, with less i-motif formation, while the presence of a stable i-motif increased mRNA levels. The ability for i-motif formation to elevate gene expression that was then mitigated by resolution of the structure indicates that, in the *BCL2* promoter, the i-motif has the potential to activate transcription. 

In the companion study, discovery of the transcription factor HNRNP LL binding to the *BCL2* i-motif and enhancing mRNA expression further supported the role of the i-motif as a transcriptional activator and provided a potential mechanism [47]. HNRNP LL recognizes the lateral loops of the folded i-motif and initiates unfolding of the structure upon binding, consequently leading to increased transcription (Figure 2A,B). When the *BCL2* i-motif is already in a partially unfolded or hairpin state, this interaction cannot occur and gene expression is downregulated (Figure 2C). 

Of note, HNRNP LL is not the only HNRNP family member involved in i-motif driven mechanisms for regulating transcription. HNRNPK is known to recognize and bind i-motif structures, including in the *KRAS* promoter [49]. Similar to the interaction between the *BCL2* i-motif and HNRNP LL, destabilization of the *KRAS* i-motif disrupts the interaction between the transcription factor and the structure, causing downregulation of *KRAS* expression (Figure 2D). 

In addition to mammalian cells, the i-motif was shown to activate transcription in the invertebrate Bombyx mori, commonly known as the silkworm moth [50]. The transcription factor gene *BmPOUM2*, which coordinates a gene expression program critical for proper wing disc formation during metamorphosis, contains an i-motif-forming sequence within its promoter region. This i-motif element was characterized to form a stable structure and destabilization of the i-motif with either non-cytosine base substitutions, hybridization with an anti-sense oligonucleotide, or a small-molecule ligand decreased transcription of *BmPOUM2*. Treatment with anti-sense oligonucleotides against the complementary G4-forming sequence to prevent G4 formation confirmed that this effect was due to the formation of i-motifs rather than G4s. To further establish the regulatory role of the i-motif, a luciferase reporter assay showed that treatment with the porphyrin compound TMPyP4, which destabilizes the *BmPOUM2* i-motif, significantly reduces *BmPOUM2* transcription. This compound, detailed in Section 4, also interacts with G4 structures. While interaction with the *BmPOUM2* G4 was not discussed so its effect cannot be attributed solely to i-motif destabilization, the results of this assay confirm that the secondary structures formed in this sequence regulate gene expression [50]. Consistent with mammalian counterparts, a nuclear protein, BmILF, was identified to bind to the *BmPOUM2* i-motif and enhance promoter activity. The corroborating data across species are suggestive of a generalizable rule that i-motifs positively regulate gene expression. However, as with other cis-regulatory elements, inherent differences across promoters in the distance of the i-motif sequence from the transcription start site, composition of flanking nucleotide motifs, and the i-motif sequence itself, most likely confer promoter-specific variations on i-motif function. 

### 3.2. Transcriptional Repressor

In the context of the *c-MYC* promoter, the relative position of the i-motif-forming sequence to a transcription factor binding site alters the protein–DNA interaction and we see how the i-motif structure can act to repress transcription. *c-MYC* is one of the most frequently perturbed oncogenes in human cancers, with over 40% of tumors showing overexpression at the protein level. The cancer-enabling properties of the c-MYC protein come from its activity as a “master regulator” to propagate expression of multiple pathways associated with hallmarks of cancer, such as cell cycle signaling, and glycolysis and other metabolic cascades [51,52,53]. The expression of c-MYC is required for normal cell growth, tissue development, and apoptosis, but levels must be downregulated when growth and proliferation are complete, allowing for appropriate differentiation and senescence [54]. Because c-MYC affects the expression of a myriad of genes involved in these processes, the expression of c-MYC must be tightly regulated to prevent the development of cancers. The presence of an i-motif-forming sequence within the *c-MYC* promoter caused researchers to immediately hypothesize about its potential role in transcriptional regulation and subsequent studies show that the *c-MYC* i-motif can modulate *c-MYC* expression. 

In keeping with the theme of HNRNP proteins recognizing i-motif sequences as observed for *BCL2* and *KRAS*, HNRNPK also interacts with the *c-MYC* i-motif for transcription control. Although a seminal study from 1996 established the HNRNPK transcription factor bound to the C-rich segment within the *c-MYC* promoter and this interaction is required for activating *c-MYC* expression, this sequence was not yet known to form an i-motif structure [55]. Surprisingly, in contrast to the *BCL2* i-motif, formation of the *c-MYC* i-motif leads to lower transcriptional activity than when unfolded [56]. While i-motif formation is still necessary for initial HNRNPK recognition of cytosine tracts in the lateral loops, this interaction is not strong enough to fully activate transcription. Increasing superhelicity unwinds the i-motif and exposes additional cytosine tracts to HNRNPK. These additional elements increase the binding strength of HNRNPK sufficiently to activate high levels of transcription [56]. In this case, the i-motif plays a role in the initial binding site recognition. However, the persistence of the i-motif prevents access to the primary site for maximum transcriptional activation by HNRNPK and therefore has potential to also act as a repressor of transcription. This model of transcriptional regulation is further supported by the later use of small-molecule stabilizers [57], which are discussed below in Section 5.1.1 (Figure 2E). 

## 4. Identification of i-Motif Interactive Small Molecules 

Small molecules, including some of those discussed in Section 3.1, were critical molecular tools in defining the biological functions of i-motifs, particularly in the case of the *BCL2* i-motif and compounds IMC-48 and IMC-76. While these i-motif interactive compounds were used to elucidate the function of i-motifs, the identification of such compounds also establishes the i-motif as a therapeutic target and will facilitate drug discovery. The porphyrin, TMPyP4 (Figure 3), a previously validated pan-G4-binding compound, was the first i-motif interactive small molecule identified in 2000 [58]. Initially, TMPyP4 was found to interact with i-motif-forming sequences from human telomeres and induce i-motif formation [59]. After further study, it became increasingly clear that similar to interactions with G4s, the effect of TMPyP4 on i-motif formation varies depending on the structure. For instance, TMPyP4 increases the stability of the *c-MYC* i-motif and prevents binding of the transcription factor HNRNPK [60], but decreases the stability of the *BmPOUM2* i-motif and leads to downregulation of transcription [50]. Even though extensive structure–activity relationship analyses are lacking, i-motif destabilizing compounds (Figure 4) tend to consist of more complex, multicyclic ring systems relative to i-motif stabilizers (Figure 3). Beyond the chemical features of the ligands, what is striking about the i-motif destabilizers identified thus far is their ability to function dually as strong G4 stabilizers. These compounds are discussed in Section 4.5 below. Regardless of effect on the structure, i-motif interactive compounds tend to contain either a quaternary amine or a highly basic amine that most likely is ionized at physiological pH and presumably interacts with the negative DNA phosphate backbone to anchor the molecule in the DNA. In this section, we will discuss commonly used techniques as well as newer approaches that effectively discovered both i-motif stabilizing and destabilizing agents.

### 4.1. Fluorescence Resonance Energy Transfer (FRET)-Based Screen

Many i-motif-binding molecules are identified using a FRET-based screen, including the *BCL2* i-motif interactive small molecules IMC-76 and IMC-48 [46]. In a FRET screening assay, the i-motif sequence of interest serves as the FRET probe, with a fluorescent donor molecule attached to the 5′ end and an acceptor, or quencher, molecule to the 3′end [61]. Probes are suspended in a buffer conducive to i-motif formation, typically sodium cacodylate as it maintains a constant pH despite variations in temperature [62], and then incubated with compound libraries. The probe–ligand mixtures are exposed to a light source at the excitation wavelength of the donor fluorophore. If the i-motif structure is folded, emission from the donor fluorophore is absorbed by the acceptor fluorophore due to the close proximity of the fluorophore-labeled ends of the probe and results in a loss of fluorescence at the donor emission wavelength. Conversely, if the i-motif is unfolded, the donor and acceptor/quencher fluorophores are spatially distant and the emission wavelength of the donor fluorophore is not transferred. Thus, a compound that yields a decrease in fluorescence indicates a stabilizing interaction while an increase in fluorescence at the emission wavelength of the donor indicates a destabilizing effect on the i-motif. A melting assay is sometimes applied to this FRET screen for i-motif interactive compounds [63]. This technique uses the same principle of donor and acceptor fluorophores, but measures fluorescence across a range of increasing temperatures to determine the thermal stability inferred by the melting temperature at which half the signal is lost. In this case, increased melting temperature indicates an increase in structure stability. When using FRET to screen for interactive compounds, it is important to use a secondary method to confirm interaction such as circular dichroism spectroscopy (CD) or UV spectroscopy as some molecules can interact with the fluorophores attached to the probes or the fluorophores themselves can alter the stability of i-motif structures [64].

### 4.2. Fluorescent Intercalator Displacement (FID) Assay 

In recent years, other approaches successfully identified i-motif interacting compounds without the need for fluorescently labeled i-motif probes. One such method is the FID assay [65]. FID assays are routinely employed to discover compounds that bind to various DNA structures and recently the technique was adapted for the i-motif using the fluorescent probe thiazole orange [66]. Thiazole orange is not fluorescent until it binds to DNA, and when thiazole orange binds to an i-motif the compound exhibits detectable fluorescence. Thiazole orange is considered ideal for this assay because of the high affinity for i-motif DNA that is strong enough to create an observable fluorescent signal, but still allows for displacement by other i-motif interacting ligands to create a fluorescence detection window for stabilizers. Potential i-motif interacting compounds are screened by adding compounds to a solution containing thiazole orange already bound to the i-motif oligomer of interest and observing changes in fluorescence. The loss of thiazole orange fluorescence intensity identifies molecules that bind to the i-motif with higher affinity. Development of this assay for i-motif-binding ligands led to the discovery of compounds mitoxantrone, tobramycin, tilorone, Harmalol, and quinalizarin, which interact with telomeric and *c-MYC* i-motifs [66].

### 4.3. Electrospray Ionization Mass Spectrometry (ESI-MS)

Similar to most assays utilized to study the i-motif, a rapid ESI-MS screening assay was originally adapted to detect G4 interactive ligands [10]. This method is recognized as equally effective at screening small molecules for i-motif interaction [67]. The ESI-MS is combined with capillary electrophoresis to create a rapid, automated screening process that uses a minimum amount of sample. A low input is possible because, rather than evaluate molecules in solution, ESI-MS involves conversion of molecules from a liquid solution into a gas phase [68]. This process allows for a highly sensitive determination of i-motif formation and assessment of stability in addition to providing information about ligand binding interactions. ESI-MS coupled with capillary electrophoresis is a valuable tool for screening potential i-motif interactive compounds that provides more data regarding binding strength and stoichiometry in comparison to many other methods. The drawback to the ability of assaying minimal oligomer and ligand material is that ESI-MS cannot evaluate ligand binding in solution, which is necessary to determine the kinetics of the interaction and the folding/unfolding of the i-motif structure in response to ligand binding. Thus, while this assay serves as an efficient first-screening method to save on reagents and generate a short ‘hit’ list of compounds, follow-up assays, such as CD and NMR, are necessary to validate these compounds as i-motif interactive agents in solution.

### 4.4. Small-Molecule Microarrays (SMMs)

The final screening technique we will discuss is the SMMs, which efficiently supports high-throughput screening of small compounds. SMMs were developed to screen for molecules that bind to various proteins and effectively adapted for nucleic acid applications [69]. In this assay, collections of thousands of small molecules are immobilized as spots on glass slides arrayed into rows that are subsequently probed with a fluorescently tagged i-motif-forming oligomer of interest [70]. Under conditions for inducing i-motif formation, slides are incubated with the labeled i-motif and then through a series of wash steps, non-specific, low-affinity interactions are rinsed away. A microarray scanner detects spots with fluorescent activity that correspond to compounds with potential to bind to the i-motif. This technique is highly successful for detection of G4 ligands and, subsequently, SMMs were used to identify candidate small molecules that interact with the i-motif within the *HRAS* oncogene promoter [71]. To confirm that the compounds interact with the folded i-motif rather than duplex or hairpin formations, the experiments were conducted at various pH levels and only compounds that demonstrated pH-dependent binding were considered for further study. While SMMs are not yet widely used to identify i-motif-binding ligands, the high-throughput capabilities of this method make it an attractive option for studying i-motifs as therapeutic targets. 

### 4.5. G4 Interaction as an Important Consideration for Identifying i-Motif Ligands

Since i-motifs were discovered later than G4s and only recently pursued as potential targets for therapeutics, many reported G4 stabilizing compounds were not tested on the complementary i-motif structures. In 2018 and 2019, however, two studies were published examining the effects of G4 stabilizers on i-motifs. The 2018 study treated oligomers of the telomeric i-motif sequence with six known G4 stabilizers: berberine, BRACO-19, mitoxantrone, phen-DC3, Pyridostatin, and RHPS4 (Figure 3 and Figure 4) [64]. All six compounds proved to bind to the i-motif via FID assay and three of them (BRACO-19, Mitoxantrone, and Phen-DC3) caused destabilization of the i-motif structure as measured by changes in thermal stability. Although FID assays showed that the other compounds bound to the i-motif, subsequent experiments showed that these interactions had no effect on structure stability. In the 2019 paper, the same compounds along with TmPyP4 were tested on i-motif sequences from promoter regions that form structures at neutral pH, death-associated protein (DAP) and ataxin type 2-related protein (ATXN2L) [72]. Again, all compounds were shown to interact with these two promoter i-motif structures. In the case of the DAP i-motif sequence, the small molecules destabilized the structure. For the ATXN2L i-motif, only berberine, BRACO-19, phen-DC3, and RHPS4 lowered the stability of the structure, while mitoxantrone, pyridostatin, and TmPyP4 stabilized the i-motif as measured by an increase in the thermal stability. The authors hypothesized that the stabilization of the ATXN2L i-motif by these compounds was likely due to stabilization of an alternate secondary structure or a change in the unfolding kinetics, leading to the higher melting temperature. These studies not only emphasize the importance of considering the effects on i-motif structures when developing G4 ligands, but they also demonstrate the possibility of simultaneously targeting both G4s and i-motifs with a single ligand in order to modulate gene expression. This multi-targeted approach could be beneficial when designing therapeutics to treat drug-resistant cancers.

## 5. Therapeutic Potential of Targeting the i-Motif in Cancer

i-Motif sequences are prevalent in promoter regions, including those of oncogenes (Table 1). Given the evidence that i-motifs act as transcriptional regulators, these structures are ideal targets for shutting down oncogenic signaling. Oncogenes are normal genes (proto-oncogenes) that lead to the initiation and/or progression of cancer when aberrantly activated due to mutations or other genomic events. i-Motifs are also present in telomeres, where stable i-motifs are shown to block the progression of telomerase. Dysfunctional telomeres and abnormal telomerase activity prolong cell survival, a critical hallmark of cancer, and increase the likelihood of accumulating oncogenic mutations with the ability to bypass apoptotic cell death. Thus, pharmacological targeting telomeric i-motifs present another avenue for therapeutic intervention in cancer. 

### 5.1. Stabilization of the c-MYC and Telomeric Structures Inhibits Tumor Growth

#### 5.1.1. *c-MYC*

As discussed in Section 3.2, the *c-MYC* promoter contains an i-motif-forming sequence that plays a role in its transcriptional regulation. While c-MYC protein is important for normal cellular function in many cell types, when mutations eliminate the reliance of c-MYC activation on growth factor signaling, this oncogene helps drive cancer initiation and progression. Small-molecule stabilizers of the *c-MYC* i-motif that decrease expression of this key oncogene have immense utility as new agents for cancer treatment especially given that the c-MYC protein is considered undruggable. Recent efforts to develop therapeutics against this well-sought-after molecular target from researchers at Sun Yat-sen University in China led to the identification of promising compounds that stabilize the *c-MYC* i-motif [57,73]. In 2018, they identified an acridone derivative, 2-methylacridin-9(10*H*)-one, as a *c-MYC* i-motif stabilizer and generated analogs to increase selectivity [57]. While multiple compounds were found to increase i-motif stability in comparison to the parent compound, B19 stood out as a prime candidate (Figure 3). This molecule displayed strong binding affinity and considerable preference for the *c-MYC* promoter i-motif structure over G4 and duplex DNA. Compound B19 not only stabilized the i-motif, but induced folding of the structure from a linear conformation. In confirmatory assays, compound B19 significantly reduced transcriptional activation as demonstrated in a dual-luciferase reporter assay. B19 also lowered mRNA and protein expression, reduced the proliferation of cervical cancer cells through inducing apoptosis, and prevented metastasis in vitro [57]. 

Two years later, the same group discovered a bisacridine derivative, compound a9 (Figure 3), that targets both the *c-MYC* i-motif and G4 structures with higher selectivity than duplex and hairpin DNA [73]. They also showed that compound a9 preferentially stabilizes the *c-MYC* i-motif and G4 over secondary structures from promoter regions of other genes. Relative to compound B19, a9 exhibited high potency at nanomolar to low micromolar concentrations in effectively reducing c-MYC mRNA and protein levels. Compound a9 inhibited proliferation in multiple cancer cell lines, including those derived from cervical, lymphoma, lung, bone, colon, and liver cancers, and tumor growth in an in vivo model of cervical cancer. Notably, compound a9 was not toxic to the mice in terms of overall weight loss or changes in organ weight compared to cisplatin (a mainstay of cervical cancer therapy), which caused significant reductions in body weight and weight of the liver and spleen. Compounds that target both i-motifs and G4s are of particular interest because the development of drug resistance is the primary obstacle to achieving successful treatment of many human cancers. As stabilization of the *c-MYC* G4 is known to also block transcription [74], stabilizing both transcription regulators with a single drug could minimize drug resistance. 

#### 5.1.2. Telomeres

When a cell duplicates its DNA, the DNA polymerase fails to replicate to the very ends of chromosomes such that with each cellular replication cycle, chromosomes are shortened. To prevent premature shortening and protect the valuable, internal genetic information from this gradual erosion, the DNA at chromosome ends, referred to as the telomeres, consist of TTAGGG/AATCCC repeats that are elongated during DNA replication by the telomerase enzyme [75,76,77]. However, over the lifetime of a cell, telomeres reach a critical length and programmed cell death is initiated. In some cancers, this process is hijacked with higher than normal expression or activity of telomerase, permitting a continuous extension of telomeres, which contribute to the sustained proliferation and immortalization tumor phenotypes [77]. Accordingly, telomerase inhibition offers a reasonable targeted approach to hinder tumor growth. With the G/C-rich telomere sequences comprising some of the most well-studied G4 and i-motifs [78,79], the stabilization of these structures is an active area of drug development in the field. The first reports that stabilization of G4s with small molecules inhibited telomerase activity started in the late 1990s [58], while telomeric i-motif stabilization was not demonstrated until 2012.

The first compounds shown to selectively stabilize the telomeric i-motif and inhibit telomerase were carboxylated single-walled carbon nanotubes (SWNTs) [80]. SWNTs can enter cells through the lipid bilayer and accumulate in the nucleus, where they bind to the major groove of DNA and induce stable formation of i-motif structures. This effect is unique to i-motifs, with no effect observed on the formation or stability of G4s. In vitro assays showed SWNT-induced stabilization of telomeric i-motifs directly leads to the loss of telomerase activity, which was then confirmed in human cancer cells. It is important to note, SWNT treatment was not immediately cytotoxic. There was a two-week delay before observing a significant decline in cellular proliferation with an additional week until proliferation ceased altogether. i-Motif stabilization by SWNTs causes the uncapping of telomeres and induces a DNA damage response localized to the telomeres that eventually initiates cell cycle arrest, apoptosis, or senescence. Subsequent evidence suggests that the mechanism for SWNT stabilization of i-motifs involves the proton exchange between the carboxyl group on the SWNT and the i-motif cytosines, resulting in hemi-protonation of the i-motif-forming sequences and folding of a stable structure [81]. SWNTs have received attention as potential drug delivery devices and their ability to inhibit telomerase via i-motif stabilization and induce anti-proliferative effects hints at an additional intriguing potential strategy for future cancer therapeutics [80].

### 5.2. Destabilization of the KRAS and PDGFR-β i-Motifs Blocks Oncogenic Signaling 

#### 5.2.1. *KRAS*

In healthy cells, the protein KRAS, a member of the RAS family of GTPases, acts as an on/off switch for cell proliferation, survival, differentiation, and metabolism signaling pathways [82,83,84]. Mutations within KRAS are often seen in human cancers and account for approximately 86% of RAS mutations [82]. These mutations prevent the hydrolysis of GTP to GDP, which disables the “off switch”, constitutively activating KRAS regulated signaling pathways that drive tumor growth [85]. There is also evidence that KRAS mutations are involved in tumor cell evasion of host immune responses [86]. Similar to c-MYC, KRAS was considered impossible to target for many years. The active site of KRAS consists of small and transient allosteric areas that make designing inhibitory drugs difficult [87]. Although development of small molecules to inhibit KRAS has progressed, targeting *KRAS* at the gene level still remains a more attractive strategy [83].

Along with *BCL2* and *c-MYC*, the i-motifs in the *KRAS* promoter are among the more thoroughly investigated. Three potential i-motifs have potential to form within the *KRAS* promoter with the most stable structure arising from the mid-region sequence [49]. The mid-region i-motif is also the structure to which the transcription factor HNRNPK binds, resulting in transcriptional activation. The same study discovered a benzophenanthridine alkaloid, nitidine (Figure 4), from a FRET screen that binds to and destabilizes the mid-region i-motif as well as prevents interaction between HNRNPK and the i-motif. Nitidine seems selective for the KRAS i-motif structure since there was no measurable effect on the stability of the *BCL2* or *c-MYC* i-motif structures. The mid-region KRAS i-motif contains a long loop region that forms a short hairpin structure. It was proposed that this additional hairpin structure may be important for HNRNPK binding and that nitidine preferentially acts on the KRAS i-motif due to this hybrid structure. Thus, nitidine interaction with other i-motifs that form similar hairpins within their loops cannot be ruled out. Treatment with nitidine downregulated *KRAS* mRNA production in the pancreatic cancer cells; however, whether this persisted through to the protein level or altered cell proliferation is not known. While there are unanswered questions regarding the potential of nitidine as a lead compound for therapeutic development, this initial study provides evidence for targeting *KRAS* expression through modulating i-motif stability. 

#### 5.2.2. PDGFRβ

PDGFRβ is a tyrosine kinase receptor that activates Ras-MAPK, PI3K, and PLC-γ growth and calcium-dependent transduction pathways [88,89]. High levels of PDGFRβ expression and activity through different mechanisms including gene translocations are implicated in solid, epithelial-based cancers and vascular diseases [90,91]. Cells overexpressing the receptor can release PDGFRβ ligands, propagating an autocrine signal loop that leads to proliferation of these unstable cells and contributes to tumor development. These ligands are also known to recruit stromal cells to the tumor through paracrine signaling [92,93]. While there are therapies that target the PDGFR-β protein, including tyrosine kinase inhibitors such as imatinib and neutralizing antibodies against PDGFR-β [94], there is still a need for more effective drugs to inhibit aberrant signaling through this receptor [91]. 

In 2017, Brown et al. published a study targeting transcription of PDGFRβ through G4s within the complex nuclease hypersensitive element in the promoter region in order to prevent its expression [95]. This sequence can form four different G4s, with the 3′ end G4 structure responsible for repression of transcription. To define this repressor effect, they created oligomers with three different point mutations within the 3′ end G4 sequence. Mutation of the 3′ adenine to a guanine resulted in a drastic increase in G4 stability. Paradoxically, the stabilization of the repressor G4 increased transcription. The counterintuitive result incited attention on the i-motif sequence and the corresponding thymine to cytosine transition. The additional cytosine within the i-motif-forming sequence stabilized the i-motif and attributed to the transcriptional activation. Comparison of the wild-type i-motif sequence, which is capable of forming at least two i-motifs in equilibrium, led to the discovery that the mutant sequence is locked into forming a single, highly stable structure [95]. Screening the NCI Diversity Set library identified benothiophene-2-carboxamide (NSC309874; Figure 4) as a destabilizer of both the wild-type and mutant i-motifs. NSC309874, which did not significantly interact with the *PDGFRβ* G4 structures, repressed *PDGFRβ* transcription in an aggressive neuroblastoma cell line indicating the potential of this compound as a new *PDGFRβ* inhibitor. 

### 5.3. Potential of Targeting i-Motifs in Other Cancer-Related Genes

In addition to the cancer-enabling genes with defined i-motifs discussed above, there are several other strong candidates as future drug targets worth mentioning (Table 1). The precise function of the i-motifs shown to form in the genes listed below is not fully teased out, but considering the growing body of evidence that i-motifs are modulators of gene expression, it is likely the structures found in these genes also regulate transcription. 

#### 5.3.1. i-Motifs in Oncogenes 

While the characterization of the *VEGF* i-motif [96] was a part of the early cohort of oncogenes, i-motifs in the *BRAF* [97], *c-MYB* [98], and *HRAS* [99] oncogenes were only detected in the last five years. Both the BRAF kinase and the HRAS GTP hydrolase promote RAF/MEK/ERK signaling and activating mutations in either protein causes constitutive signaling and subsequent uncontrolled growth and proliferation [100,101]. Such mutations occur in many cancers, but are especially common in melanoma for BRAF [102] and head and neck, bladder, and thyroid cancers for HRAS [103]. c-MYB is a transcription factor that controls expression of genes involved in hematopoiesis and colonic crypt formation, and may also play a role in tumor immune surveillance in natural killer cells [104,105]. Oncogenic mutations within c-MYB are associated with multiple cancer types including pancreatic, colon, prostate, and liver cancers [106]. The *VEGF* oncogene is actively pursued as a cancer target with already FDA-approved inhibitors (sorafenib, sunitinib, and bevacizumab) that shut down this primary driver of angiogenesis [107,108]. The flavonol fisetin (Figure 4) destabilizes the *VEGF* i-motif, potentially binding preferentially to the alternative hairpin structure, but the biological role of this i-motif and the effects of its destabilization on *VEGF* transcription remain unclear [109].

#### 5.3.2. i-Motifs in Tumor Suppressor Genes

The enrichment of G4 and i-motif-forming sequences within oncogene promoter regions [11,12,45] naturally steers drug discovery efforts towards oncogenes. Furthermore, in light of research characterizing G4 structures as transcription repressors, the ability to turn off gene expression is seemingly more attainable from a drug development perspective than turning on expression, which is needed in the case of restoring tumor suppressors. Based on data supporting a switch-like function of i-motifs for modulating transcription [46], targeting these structures in tumor suppressor promoters is a feasible approach to reactivate the lost cellular guardians. Thus far, *DAP* [45,71], *RAD17* [110,111], *Rb* [112], and *SMARCA4* [113] tumor suppressor genes are reported to contain i-motif-forming elements within their promoters. Reduced expression of DAP promotes cell migration and tumor growth and is correlated with the pathology of breast and colon cancers [114,115]. Loss of RAD17 is implicated in colorectal [116], non-small-cell lung [117], and head and neck cancers [118]. RAD17 normally suppresses genomic instability by maintaining the integrity of DNA repair and cell cycle checkpoint prior to DNA replication in the S phase [119,120]. Similarly, Rb is a negative regulator of cell cycle progression. Mutations and deletions of Rb are found in many cancers [121,122,123,124]. Interestingly, SMARCA4 acts primarily as a tumor suppressor, as its loss leads to inactivation of the Rb pathway in ovarian and non-small-cell lung cancers, but is also considered an oncogene in other contexts [125,126,127,128]. Overexpression of SMARCA4 in breast and late-stage pancreatic drives tumor cell proliferation and is associated with progressive disease and poor prognosis [129,130,131]. The i-motif upstream of the *SMARCA4* promoter is moderately stabilized by TMPyP4 [113], but to date no studies seek to determine the effect of i-motif stabilization on *SMARCA4* expression.

#### 5.3.3. i-Motifs in Other Cancer-Related Genes 

Additional genes related to propagating cancer cell phenotypes that do not fit into either the oncogene or tumor suppressor categories were recently discovered to harbor i-motif-forming sequences capable of adopting structures. Acetyl CoA Carboxylase 1 (ACC1) is the control enzyme for fatty acid metabolism [132]. Mutations in the oncoprotein BRCA1 can activate ACC1 and perturb regulatory mechanisms, allowing cancer cells to synthesize fatty acids more quickly [133]. Many cancer types exhibit ACC1 overexpression enhancing migration and invasion in breast cancer [134] and proliferation of cancer stem cells [135]. An i-motif structure that forms from the C-rich *ACC1* promoter sequence inhibited transcription in a luciferase assay, whereas a mutant sequence that prevented i-motif assembly increased transcription [136]. While this work nicely demonstrates formation of a novel i-motif, a follow-up study is needed to assess whether the effects on gene expression were due to loss of the i-motif or changes to the complementary G4 [136].

*ATXN2L*, which was also recently discovered to contain an i-motif-forming sequence, encodes a key component of stress granules. During times of cellular stress, granules containing RNA-protein complexes are formed in the cytoplasm in response to impaired translational initiation [137]. Overexpression of ATXN2L leads to excessive formation of stress granules and reduced expression prevents their formation [138]. Dysregulation of ATXN2L-dependent stress granule formation is seen in gastric cancer and T-cell lymphomas and can promote cell survival, proliferation, and metastasis [139,140]. 

## 6. i-Motif-Targeting Compounds as Potential Therapies for Non-Cancer Human Diseases

**Figure 5:**
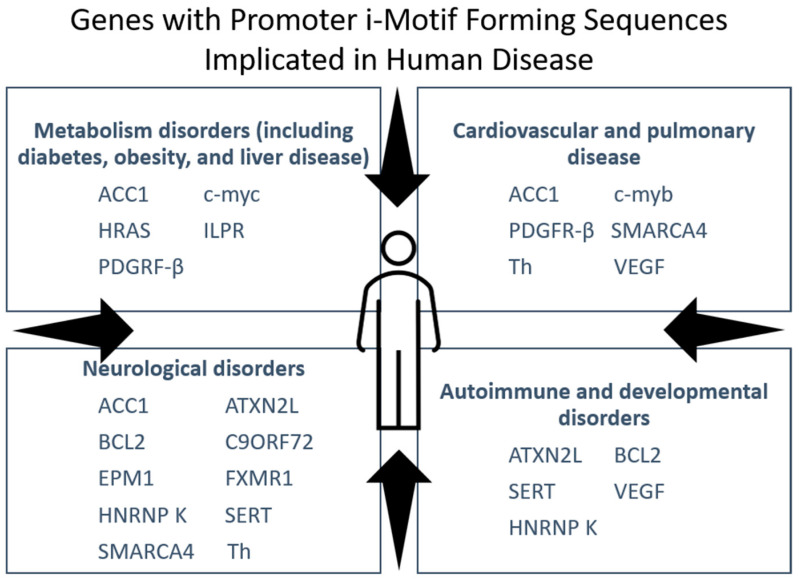
i-Motif sequences are found in the promoters of genes implicated in many human diseases besides cancer.

Numerous human diseases are linked to a particular defect in the cell genotype. Besides genes integral to the pathology of cancer, i-motif sequences are found in areas of the genome associated with other diseases (Figure 5). Certain genetic diseases, such as fragile X syndrome, stem from expanded guanine and cytosine-rich (G/C) repeats in 5′ untranslated regions capable of folding into i-motif structures. This section examines the current literature on i-motifs that form within key genes in liver disease and select neurological disorders, although we acknowledge there are other illness such as diabetes with recognized connections to i-motif-containing genes. 

### 6.1. Liver Diseases 

Non-alcoholic fatty liver disease (NAFLD) and non-alcoholic steatohepatitis (NASH) are both characterized by liver injury and inflammation, which are driven in part by excessive apoptosis of hepatocytes due to low BCL2 expression [141,142]. Previous studies establishing the i-motif within the *BCL2* promoter as a transcriptional activator [46,47] support the premise that stabilization of the *BCL2* i-motif in NAFLD and NASH is an effective approach to increase BCL2 levels and prevent hepatocyte apoptosis. Ding Li’s laboratory identified an acridone derivative, A22, as a high-affinity stabilizer of the *BCL2* i-motif (Figure 3) [143]. In order to demonstrate preferential selectivity, they tested the compound against the *BCL2* G4, duplex DNA, and several other i-motif sequences. Moreover, treatment of hepatocytes, grown in palmitic acid oil to mimic the lipid-induced apoptotic environment of NASH, with A22 resulted in a robust increase in cell viability and a decrease in activation of the apoptotic cell death pathway. Since NAFLD and NASH are tightly linked to metabolic syndrome, the authors also examined the effect of A22 treatment on glucose uptake and demonstrated that A22 was significantly more efficacious in stimulating the influx of glucose into hepatocytes compared to the metformin, the frequently prescribed treatment for insulin resistance [143]. Compound A22 also performed well in a NAFLD/NASH mouse model where treatment led to elevated BCL2 levels, less apoptosis, and decreased markers of hepatocyte injury and metabolic syndrome (e.g., weight gain not associated with food intake, insulin resistance, and elevated serum cholesterol and triglycerides). In view of these data, compound A22 and increasing BCL2 expression is a promising therapeutic strategy for diseases where cell survival is compromised, including NAFLD/NASH. Caution is warranted, of course, for approaches that aim to upregulate proto-oncogenes. Treatment with A22 also elicited a dose-dependent increase in BCL2 mRNA and protein in a hepatocellular carcinoma cell line [143], emphasizing the need for further work to fine tune dosage and define the appropriate patient subsets with a favorable risk–benefit ratio to avoid development of therapy-induced malignancies.

### 6.2. Genetic Neurological Disorders Linked to Nucleotide Repeat Expansions

Nucleotide repeats, or microsatellites, are short, simple repeats that occur frequently in the genome, typically in non-coding regions, and are evolutionarily conserved [144]. Expansion of these nucleotide repeats are a type of mutation that results from amplification of these short repeats. When nucleotide repeat expansions occur in repeats with high G/C content, the elongated sequences have potential for G4 or i-motif structure formation [145]. i-Motifs and G4s facilitate DNA polymerase stalling during replication and cause the polymerase to dissociate from the DNA [146]. When the DNA polymerase initiates replication after this dissociation, incorrect realignment along the highly repetitive DNA often occurs and results in expansion of the segment. Depending on the location, the extra stretches of repeats disrupt the reading frame for proper gene expression and increase the likelihood for secondary structure formation, which can further perturb gene replication and transcription. Nucleotide repeat expansions with repeated G/C sequences are implicated in several human diseases including fragile X syndrome (CGG), progressive myoclonus epilepsy (CCCCGCCCCGCG), and amyotrophic lateral sclerosis/frontotemporal dementia (GGGGCC) [147,148,149].

In fragile X syndrome, the loss of fragile X mental retardation 1 gene (*FRM1*) expression confers a defect in neuronal synaptic transmission and subsequent intellectual disabilities. *FRM1* is susceptible to expanded C-repeats, which are known to form i-motif structures and thought to play a role in suppressing expression of this gene [29]. A recent study revealed that the addition of a metal complex CoII(Chromomycin)_2_ causes the i-motif structures to transition into duplex DNA [147]. CoII(Chromomycin)_2_ complex preferentially binds to the duplex conformation of DNA and the authors suggest that this metal complex may prevent i-motif formation by forcing the DNA to remain in double-stranded form and, in turn, minimize polymerase slippage and inhibit further repeat expansion [147].

i-Motif formation is also observed in the relevant genomic loci of two other DNA repeat expansion-associated diseases, progressive myoclonus epilepsy [148] and amyotrophic lateral sclerosis [149]. In agreement with the *BCL2* i-motif, the i-motif-forming sequences in the genes that underlie each disease exist in a dynamic equilibrium with a hairpin [149,150]. As well, for amyotrophic lateral sclerosis, the i-motif and hairpin structures in *C9ORF72* are capable of forming even when in a duplex DNA environment, where the complimentary G-rich strand is present [150]. This preliminary work demonstrates the structural flexibility of these C-repeat regions and indicates the possibility of targeting the structures to develop new therapeutic approaches. Further research is required, however, to determine the role of i-motif formation in both the progressive myoclonus epilepsy 1 and *C9ORF72* genes in maintaining nucleotide expansion and/or transcription. 

### 6.3. Other Neurological Disorders 

A recent discovery highlights a role for i-motifs in the neurodegenerative disorder Parkinson’s disease. A 2020 study examined the activity of α-synuclein (α-syn), a protein that forms oligomers and amyloid fibrils linked to cell death of neurons [151]. While α-syn was discovered to bind telomeric G4s and i-motifs, the neuronal protein did not alter the stability of G4 structures, but promoted the folding and stability of i-motifs. Although human telomeric i-motif sequences were used as a model, the findings provide a proof of concept that α-syn may exert its cellular function through interactions with i-motifs [151]. As the mechanisms driving Parkinson’s disease are not fully defined, uncovering the interplay between α-syn and i-motifs in regulatory regions of genes critical in this disease will provide insight into the pathological defects and identify new targets for treatment.

A key pathway implicated in Parkinson’s disease and a range of other neurological conditions including bipolar disorder, schizophrenia, and addition and movement disorders is the synthesis pathway responsible for the production of dopamine, epinephrine, and norepinephrine [152]. Tyrosine hydroxylase (TH) is the rate-limiting enzyme of this pathway and contains a GC-rich element conserved across species within the *TH* promoter region capable of forming G4 and i-motif structures [153]. Interestingly, HNRNPK, the same transcription factor known to recognize and interact with the *c-MYC* i-motif sequence, also interacts with the C-rich region in the *TH* promoter in concert with cAMP Response Element-binding proteins. Consistent with promoter DNA secondary structure control of gene expression, TMPyP4 treatment of viable slices of mouse brain tissues resulted in a reduction in *TH* transcription. In further support of G4 and/or i-motif regulation of *TH* expression, a second group confirmed the ability of TMPyP4 to block transcriptional activity of the *TH* promoter [154]. This later study focused on the G4 structure and neglected the possibility of TMPyP4 perturbation of the i-motif structure, thus the predominant structure responsible for triggering the loss of transcription requires further resolution. 

In addition to the *TH* promoter, the serotonin transporter-linked polymorphic region, which regulates serotonin transporter (SERT) gene expression, another important factor in neurological diseases and psychiatric disorders, also contains an i-motif-forming sequence [155]. This i-motif structure forms at neutral pH under molecular crowding conditions and could serve as an alternative target for SERT inhibition. Selective serotonin reuptake inhibitors are commonly prescribed anti-depression and anti-psychotic medications, but cause severe adverse effects in certain patient populations [156]. As further potential for targeting the i-motif in various neurological disease states, epigenetic modifications that favor i-motif formation are hypothesized to contribute to synaptic plasticity and memory formation [157].

Lastly, there is recognition that the unknown mechanism of action of current lithium-based therapies for neurological disorders [158,159,160,161] may involve i-motif regulation as lithium cations were shown to destabilize the i-motif of telomeric C-rich oligomers [162]. Given that i-motif folding and unfolding is a relatively long process, in order to obtain robust folding kinetics by adequately capturing multiple transition states, Kim et al. developed a modified single-molecule FRET technique with extended fluorescent reads. Instead of traditional FRET, where a single fluorescent end point is analyzed, multiple fluorescence signatures are captured during the entire read period and using bioinformatics aligned together to provide an overall picture of the kinetics throughout the structural changes of the i-motif [162]. This adapted technique revealed that lithium cations destabilized the i-motif structure by enhancing the unfolding of the structure, rather than preventing the folding. Furthermore, this handshaking repeated permutation (HaRP) kinetic analysis may offer future applications to characterize interactions of i-motif ligands for therapeutic development. 

## 7. Conclusions

Over the last decade, major technological advances to detect and evaluate i-motif formation have contributed to the understanding of i-motif biology and addressed the long-standing question of whether DNA adopts such structures within cells. Whether mutual exclusive formation of the i-motif and G4 structure is a strict rule or context dependent requires further resolution. Occasionally, due to their different formation requirements or the offset position of the respective C- and G-rich sequences, both structures can form simultaneously [163]. In support of mutual exclusivity, a recent study from the Smith group demonstrates that the formation of one structure destabilizes the structure on the complementary strand [164]. Since G4s and i-motifs appear to have distinct roles in transcriptional regulation, it is reasonable that their formation within the same promoter is interdependent. From applying and modifying methods previously utilized for G4 characterization to the development of an i-motif-specific antibody that detects formed i-motifs within chromatin, molecular tools are in hand to further tease out the nuclear functions of the i-motif structure. Moving forward, additional genome-wide studies in different cellular contexts aimed at defining how i-motif formation alters the chromatin architecture and the localization of these events will aid in identifying new opportunities for therapeutic targeting. Considering the promiscuous nature of some G4 ligands to recognize the i-motif structure is also central for developing selective i-motif interactive compounds. However, perhaps achieving high levels of structure discrimination may not be necessary in genetically complex diseases such as cancer where multi-targeted approaches may provide enhanced therapeutic benefit. As research continues to delve deeper into the mechanisms behind i-motif control of nuclear processes and the structural requirements of formation within the genome, we can leverage this knowledge to design and develop a new class of targeted therapies with great potential to impact a diverse set of human diseases. 

## Figures and Tables

**Table 1:** Oncogenes, tumor suppressors, and other cancer-related genes with promoter i-motif-forming sequences.

Gene Type	Gene	Associated Cancer Type
Oncogene	*BCL2*	Colorectal, prostate, breast, lymphoma, leukemia, non-small-cell (nsc) and small-cell (sc) lung
	*BRAF*	Colorectal, gastric, thyroid, melanoma, non-melanoma skin, nsc lung
	*c-MYB*	Colorectal, gastric, esophageal, pancreatic, pediatric brain, breast, lymphoma, leukemia
	*c-MYC*	Colorectal, gastric, prostate, breast, lymphoma, leukemia, multiple myeloma, melanoma
	*HRAS*	Thyroid, cutaneous skin, lung, bladder
	*KRAS*	Colorectal, liver, pancreatic, breast, cervical, leukemia, lung, bladder
	*PDGFRβ*	Colorectal, ovarian, pancreatic, prostate, glioblastoma, breast, ovarian, various skin, lung
	*SMARCA4*	Pancreatic
	*VEGF*	Colorectal, gastric, liver, gallbladder, oral, esophageal, ovarian, pancreatic, prostate, thyroid, breast, cervical, endometrial, ovarian, osteosarcoma, nsc lung, bladder, kidney
Tumor Suppressor	*Rb*	Esophageal, pancreatic, retinoblastoma, breast, cervical, lymphoma, melanoma, sc lung, bladder
	*DAP*	Colorectal, thyroid, breast, lymphoma, lung, bladder, kidney
	*RAD17*	Colorectal, breast, nsc lung
	*SMARCA4*	Ovarian, pancreatic, uterine
Other	*ACC1*	Colorectal, liver, prostate, pancreatic, glioblastoma, other CNS, breast, leukemia, nsc lung, kidney
	*ATXN2L*	Gastric, lymphoma
